# Developmental pyrethroid exposure causes a neurodevelopmental disorder phenotype in mice

**DOI:** 10.1093/pnasnexus/pgad085

**Published:** 2023-04-25

**Authors:** Melissa A Curtis, Rohan K Dhamsania, Rachel C Branco, Ji-Dong Guo, Justin Creeden, Kari L Neifer, Carlie A Black, Emily J Winokur, Elissar Andari, Brian G Dias, Robert C Liu, Shannon L Gourley, Gary W Miller, James P Burkett

**Affiliations:** Department of Neurosciences, University of Toledo College of Medicine and Life Sciences, USA; College of Arts and Sciences, Emory University, Atlanta, GA 30322, USA; Philadelphia College of Osteopathic Medicine, Philadelphia, PA 19131, USA; Laney Graduate School, Emory University, Atlanta, GA 30322, USA; Department of Chemistry and Biochemistry, University of Notre Dame, Notre Dame, IN 46556, USA; Department of Psychiatry and Behavioral Sciences, Emory University School of Medicine, Atlanta, GA 30322, USA; Department of Medicine, University of Toledo College of Medicine and Life Sciences, Toledo, OH 43614, USA; Department of Neurosciences, University of Toledo College of Medicine and Life Sciences, USA; Laney Graduate School, Emory University, Atlanta, GA 30322, USA; Schiemer School of Psychology and Biblical Counseling, Truett McConnell University, Cleveland, GA 30528, USA; College of Arts and Sciences, Emory University, Atlanta, GA 30322, USA; Department of Cognitive Science, University of California San Diego, La Jolla, CA 92093, USA; Department of Psychiatry and Behavioral Sciences, Emory University School of Medicine, Atlanta, GA 30322, USA; Department of Psychiatry, University of Toledo College of Medicine and Life Sciences, Toledo, OH 43614, USA; Department of Psychiatry and Behavioral Sciences, Emory University School of Medicine, Atlanta, GA 30322, USA; Department of Pediatrics, Keck School of Medicine of USC, Los Angeles, CA 90089, USA; Division of Endocrinology, Children's Hospital Los Angeles, Los Angeles, CA 90027, USA; Developmental Neuroscience and Neurogenetics Program, The Saban Research Institute, Los Angeles, CA 90027, USA; Department of Biology, Emory University, Atlanta, GA 30322, USA; Center for Translational Social Neuroscience, Emory University, Atlanta, GA 30322, USA; Department of Pediatrics, Children's Healthcare of Atlanta, Emory University School of Medicine, Atlanta, GA 30322, USA; Emory National Primate Research Center, Atlanta, GA 30329, USA; Department of Environmental Health, Emory Rollins School of Public Health, Atlanta, GA 30322, USA; Department of Environmental Health Sciences, Mailman School of Public Health, Columbia University, New York, NY 10032, USA; Department of Neurosciences, University of Toledo College of Medicine and Life Sciences, USA; Department of Environmental Health, Emory Rollins School of Public Health, Atlanta, GA 30322, USA

**Keywords:** neurodevelopmental disorders, behavioral toxicology, pyrethroid pesticides, exposure models, dopamine

## Abstract

Neurodevelopmental disorders (NDDs) are a widespread and growing public health challenge, affecting as many as 17% of children in the United States. Recent epidemiological studies have implicated ambient exposure to pyrethroid pesticides during pregnancy in the risk for NDDs in the unborn child. Using a litter-based, independent discovery–replication cohort design, we exposed mouse dams orally during pregnancy and lactation to the Environmental Protection Agency's reference pyrethroid, deltamethrin, at 3 mg/kg, a concentration well below the benchmark dose used for regulatory guidance. The resulting offspring were tested using behavioral and molecular methods targeting behavioral phenotypes relevant to autism and NDD, as well as changes to the striatal dopamine system. Low-dose developmental exposure to the pyrethroid deltamethrin (DPE) decreased pup vocalizations, increased repetitive behaviors, and impaired both fear conditioning and operant conditioning. Compared with control mice, DPE mice had greater total striatal dopamine, dopamine metabolites, and stimulated dopamine release, but no difference in vesicular dopamine capacity or protein markers of dopamine vesicles. Dopamine transporter protein levels were increased in DPE mice, but not temporal dopamine reuptake. Striatal medium spiny neurons showed changes in electrophysiological properties consistent with a compensatory decrease in neuronal excitability. Combined with previous findings, these results implicate DPE as a direct cause of an NDD-relevant behavioral phenotype and striatal dopamine dysfunction in mice and implicate the cytosolic compartment as the location of excess striatal dopamine.

**Significance Statement**

A significant portion of risk for neurodevelopmental disorders (NDD) is environmental, yet very few environmental causes have been identified. Ambient exposure to pyrethroid pesticides during pregnancy has been implicated as a potential risk factor for NDD in the unborn child. Using a mouse model, we demonstrate here that low-dose developmental exposure to the pyrethroid deltamethrin causes an NDD-relevant behavioral phenotype, along with related changes in the dopamine system in the brain. These findings suggest a causal role for developmental pyrethroid pesticide exposure in NDD risk.

## Introduction

Neurodevelopmental disorders are pervasive, often incurable disorders affecting as many as 17% of children in the United States (1). Many of these disorders have few or no established biomarkers, few or no medical treatments, and can only be diagnosed behaviorally (2). For instance, autism spectrum disorder (ASD) is a cluster of complex neurodevelopmental disorders characterized by a common set of core symptoms, including persistent challenges in social interaction, communication, and restrictive and repetitive behaviors, and is frequently comorbid with other neurodevelopmental disorders such as intellectual disability, epilepsy, and attention deficit hyperactivity disorder (ADHD) (3). ASD is commonly diagnosed during childhood, when the problematic effects on the nervous system, growth, and development become apparent. Approximately 1 in 160 children worldwide and 1 in 44 children in the United States have an ASD diagnosis.

Although ASD is often considered a hereditary disorder, environmental causes contribute 38–58% of the variance in autism diagnosis (4–6). While over a hundred transgenic mouse models have been developed to investigate the genetic causes of autism (7, 8), relatively fewer environmental causes have been identified. Nonetheless, research into environmental contributors to autism risk has progressed significantly in the past decade (9). The developmental risk factors include lifestyle factors such as advanced parental age (10) and vitamin deficiency (11, 12); internal environmental factors such as gestational diabetes (13), maternal immune activation (14, 15), and prenatal hormone exposure (16); and maternal medications such as valproate (17) and selective serotonin reuptake inhibitors (18). A range of environmental chemical exposures have also now been associated with autism risk, including air pollution (19), heavy metals (primarily lead and mercury) (20), persistent organic pollutants (including DDT, polychlorinated biphenyls (21), and polybrominated diphenyl ethers) (22), and pesticides (including organophosphates and pyrethroids) (23). Nonetheless, drawing causal links between identified developmental risk factors and ASD diagnosis is challenging in human studies and highlights the critical need for animal models of exposure.

One potential environmental cause of interest is developmental pyrethroid pesticide exposure. Pesticides are biocidal chemicals commonly used as household insecticides and are critical for the agriculture industry and public health control of mosquitoes (24). Pyrethroid pesticides are becoming some of the most widely used pesticides in the United States due to their relative safety (24). Pyrethroid pesticides are nearly ubiquitous in urban streams and runoff water (25, 26), and pyrethroid exposure is so widespread that up to 70–80% of the general US population has detectable pyrethroid metabolites in their blood, despite these metabolites having a half-life measured in hours (27, 28). While low-level exposures have been deemed safe by regulators, exposure to high doses of pyrethroid pesticides adversely affects fetal development in humans and animal models (29–31).

Critically, evidence from recent epidemiology and longitudinal studies suggests that ambient prenatal exposure to pyrethroid pesticides poses a risk for autism, developmental delay, and neurodevelopmental disorders in general (23, 32–34). Analysis of data from the CHARGE study showed a significant increase in risk for either ASD or developmental delay from exposure during pregnancy to pyrethroid pesticides being applied up to 1.5 km from the home (23). A regional study in New York showed an association between areas where aerial application of pyrethroid pesticides was used, and ASD and developmental delay prevalence in the area (33). Additionally, the presence of pyrethroid metabolites in blood or urine correlates with risk for ADHD in children (27, 28).

Dopamine dysregulation is a common toxic property of environmental contaminants that may underlie the link between exposure and neurological disorders (35). In humans, dopamine and the dopaminergic signaling pathway have been implicated in the etiology of autism (36–52). In autistic individuals, alterations in reward processing for both social and nonsocial rewards have been documented (36–42), as well as disruptions in mesocorticolimbic function (42–44), dopamine release and reuptake (45, 46), and dopamine function in the prefrontal cortex and both dorsal and ventral striata (46, 47). Drugs targeting dopamine receptors are some of the most commonly prescribed treatments for comorbidities and associated symptoms in autism (51). Therefore, developmental environmental exposures that cause changes to the dopamine system might be associated with autism risk.

Recent evidence from mouse models indicates that developmental exposure to the Environmental Protection Agency (EPA) reference pyrethroid, deltamethrin (DPE), at doses far below the EPA benchmark dose lower confidence limit (10.1 mg/kg), is sufficient to cause permanent alterations to dopamine neurotransmission in dorsal and ventral striata, including altered levels of dopamine, dopamine transporter (DAT), and dopamine D1 receptor expression (27). These changes in the dopamine system are a direct cause of an ADHD-related behavioral phenotype in mice, including hyperactivity, impulsive behavior, and impaired working memory. However, it is unknown whether these ADHD-related behaviors are the only altered behaviors in developmentally exposed mice, or whether they are part of a larger behavioral phenotype.

Neither the full spectrum of behavioral changes caused by DPE nor the alterations in dopamine storage, release, trafficking, and signaling are completely understood. One critical question is whether developmental exposure to low-dose pyrethroid pesticides causes a behavioral phenotype broader than that associated with ADHD, including behaviors relevant to autism, intellectual disability, and NDD in general. Further, whether the developmental changes in striatal dopamine function are related to changes in vesicular, synaptic, cytosolic, or extracellular dopamine is unknown, and each outcome would alter the potential pharmacological and neurological treatment approaches.

To examine the effects of DPE in mouse, we exposed three independent cohorts of mouse dams to the pyrethroid pesticide, deltamethrin, at a dose well below the EPA-determined benchmark dose throughout pregnancy and lactation. We then performed behavioral and molecular assays on the resulting offspring using a litter-based design. We employed a broad behavioral battery encompassing several behavioral domains with multiple tests for each domain, including assays for social behaviors, restrictive and repetitive behaviors, communication, and cognition. Two cohorts were used as discovery cohorts for behavioral tests and a third cohort as a replication cohort. We further examined the effects of pesticide exposure on the striatal dopamine system by measuring total striatal dopamine, stimulated dopamine release and reuptake, dopamine vesicle protein markers, striatal DAT expression and function, and postsynaptic response kinetics. We hypothesized that DPE would cause an autism- and NDD-relevant behavioral phenotype and compartment-specific changes in dopamine that could be detected using these approaches.

## Results

### Cohorts

**Fig. 1.**
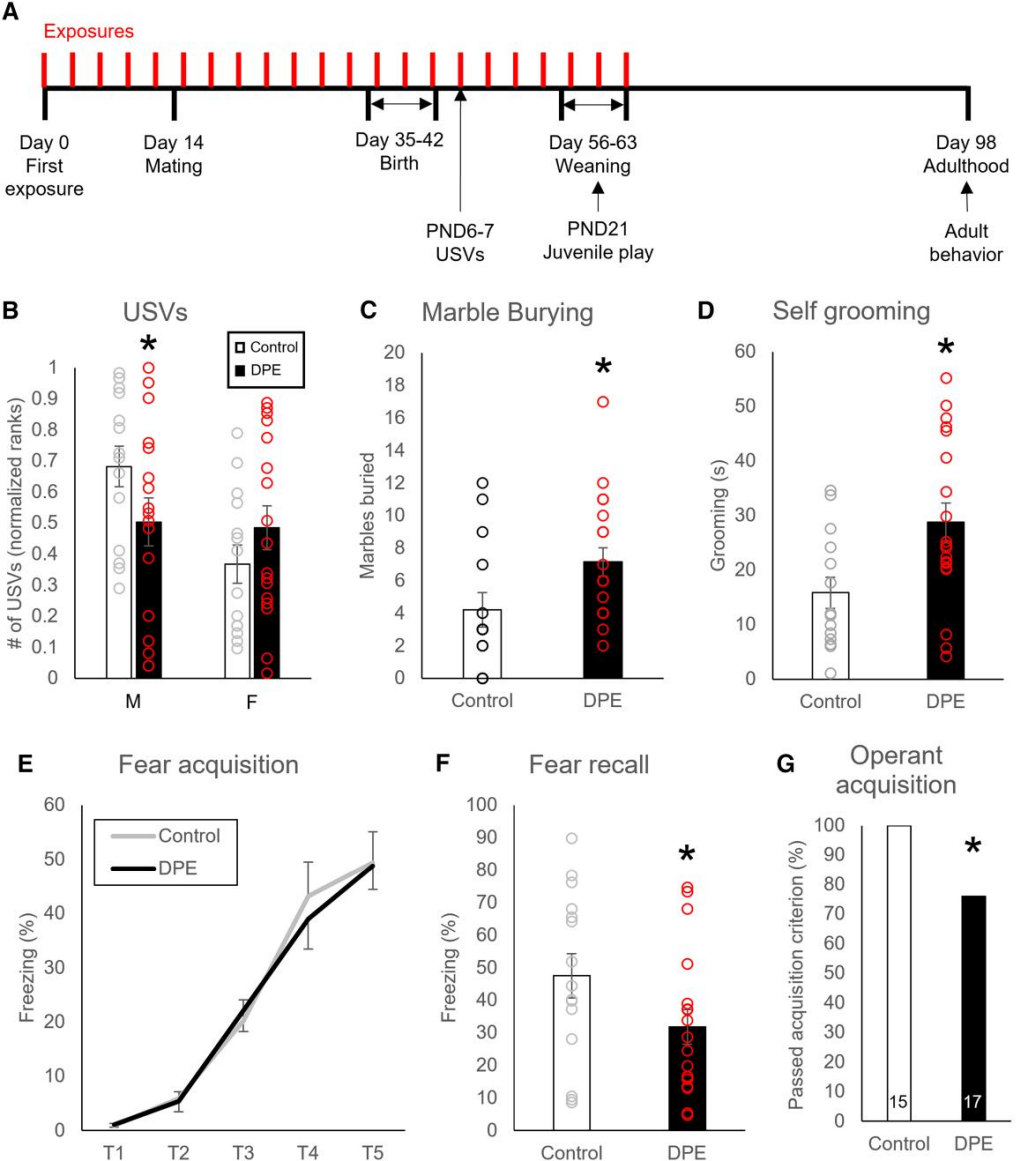
NND-related behavioral changes in DPE mice. A) The experimental design, showing pesticide exposures in the dam (3 mg/kg every 3 days) for 2 weeks preconception, then throughout pregnancy and lactation. Time points for testing of offspring are shown. B) DPE male pups produced fewer USVs than controls, while female pups were unaffected by DPE. C) DPE mice (*N* = 14) showed more repetitive digging than controls (*N* = 19) in a marble-burying test. D) DPE male mice (*N* = 14) performed more repetitive self-grooming than controls (*N* = 19) in a novel cage observation. E) DPE male mice (*N* = 15) showed no difference from controls (*N* = 18) on within-session fear acquisition. F) Following classic fear conditioning, DPE male mice (*N* = 15) showed less freezing than controls (*N* = 18) in the fear recall test. G) DPE male mice (*N* = 15) were less likely than controls (*N* = 17) to acquire the operant conditioning response to nose poke for food after 7 days of training. Error bars are SEM. *P < 0.05 by *t* test (B–F) or *χ*^2^ (G).

Exposures in the dams and behavior tests in the offspring followed the experimental timeline outlined in Fig. 1A. For behavioral experiments, the results from the discovery cohorts appear in the Supplementary material and are summarized in Table S1. The results from the replication cohort are presented here and summarized in Table S2. Results from molecular assays appear here and are summarized in Table S3.

### DPE reduced communication behavior in pups

Ultrasonic vocalizations (USVs) in mouse pups were assessed as a measure relevant to communication behavior (Fig. 1B and Table S2). Male DPE pups produced fewer vocalizations than controls during a brief separation from the dam, while females were unaffected by DPE [ANOVA, treatment by sex interaction, *F*(1,29) = 4.5, *P* = 0.043; post hoc *t* test, *P* = 0.048]. Acoustic features of pup calls were unchanged by DPE (discovery cohort, Table S1).

### DPE increased repetitive behaviors in adulthood

Marble burying and repetitive grooming/circling were used as measures relevant to repetitive behaviors (Fig. 1C and D and Table S2). Adult DPE mice buried 2–4 times as many marbles as controls (*t* test, discovery: *P* = 0.0016, replication: *P* = 0.019; Fig. 1C) and performed significantly more self-grooming during novel cage observations (Mann–Whitney *U*, *P* = 0.03; Fig. 1D).

### Social behaviors were unaffected by DPE

Various assays of mouse social behavior were used as measures relevant to social functioning. There were no observed differences between control and DPE mice on measures of social behavior taken during assays for juvenile play, three-chamber social approach, social interaction, or social fear transmission (*P* > 0.1 for all measures; see Table S1).

### DPE impaired learning and memory

Classic fear conditioning and operant conditioning were used as assays relevant to cognition (Fig. 1E–G and Table S2). For classic fear conditioning, freezing was measured during a cued fear conditioning task at baseline, during fear acquisition, and during fear recall, with freezing during tones in the fear recall test as the primary outcome of interest. There were no differences between exposure groups in baseline freezing (*t* test, *P* = 0.30) or fear acquisition [ANOVA, no tone × exposure interaction, *F*(4,124) = 0.24, *P* = 0.92; Fig. 1E], with both groups acquiring a fear response [ANOVA, main effect of tone, *F*(4,124) = 85.5, *P* < 0.0005]. Nonetheless, DPE mice showed less freezing during fear recall (one-tailed *t* test, *P* = 0.036; Fig. 1F). Separately weaned littermates not exposed to fear conditioning were trained on a fixed ratio 1 (FR1) operant conditioning task for food rewards. After 7 days of training, fewer DPE mice than control mice passed the acquisition criterion (*χ*^2^ test, *χ*^2^ = 4.0, *P* = 0.022), with 100% of control mice acquiring the operant response (Fig. 1G).

### DPE increased striatal dopamine content and release

**Fig. 2.**
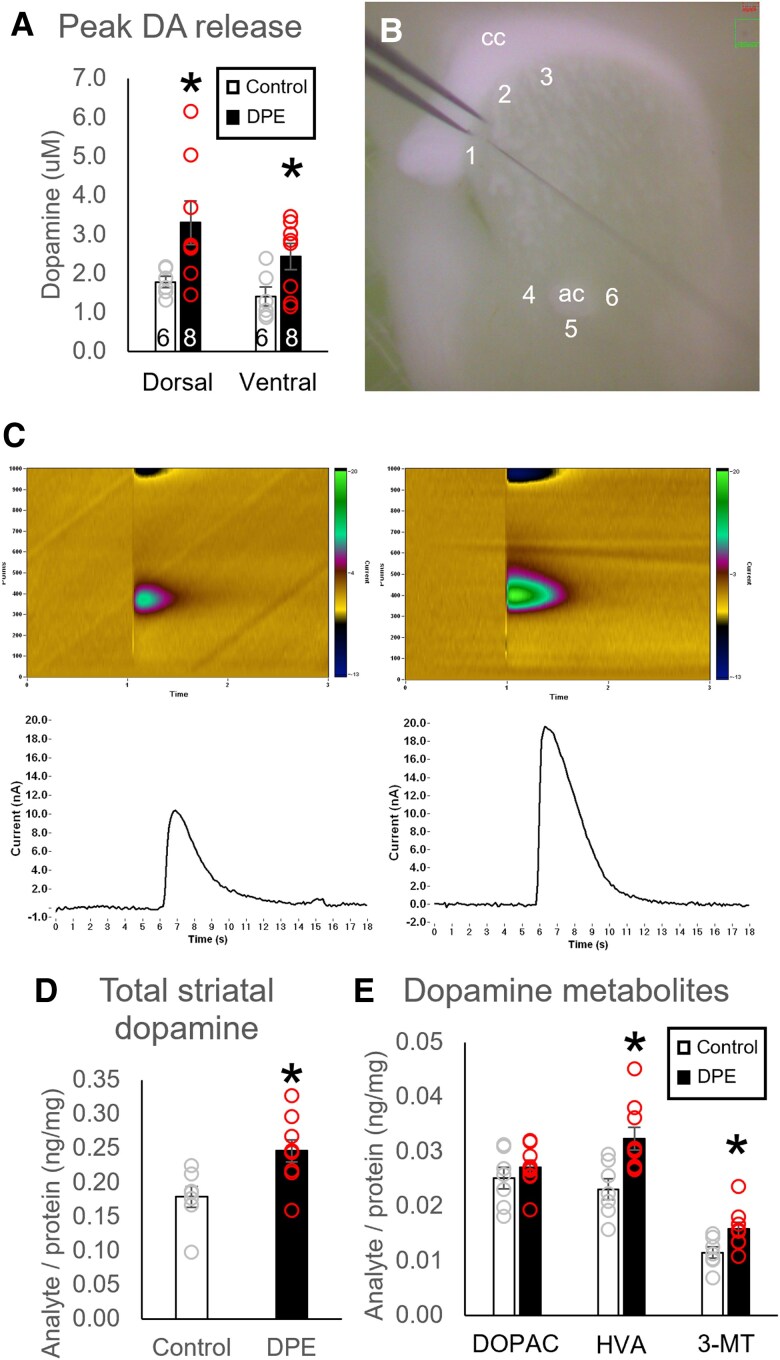
Neurochemical changes in striatal dopamine content and release in DPE mice. A) DPE mice (*N* = 8) showed a higher peak of stimulated dopamine release than control mice (*N* = 6) in both the dorsal and ventral striata. B) A representative image shows placement of the stimulating and recording electrodes for cyclic voltammetry in the dorsal striatum (sites 1–3) and ventral striatum (sites 4–6). C) Representative cyclic voltammetry color plots and amperograms for the ventral striatum of control mice (Left) and DPE mice (Right). D) Total striatal dopamine and E) two out of three dopamine metabolites were elevated in DPE mice (*N* = 9) relative to controls (*N* = 7). *P < 0.05 by *t* test. cc, corpus callosum; ac, anterior commissure.

Stimulated dopamine release in the dorsal and ventral striata was quantified using fast-scan cyclic voltammetry (Fig. 2A–C and Table S3). Compared with controls, DPE mice had higher peak striatal dopamine release in the dorsal and ventral striata following stimulation (dorsal: *t* = −2.34, *P* = 0.037; ventral: *t* = −2.29, *P* = 0.041). There were no differences between groups in measures of tau (*P* > 0.1). We then quantified dopamine, its metabolites, and its cysteinyl adducts in striatal tissue using high-performance liquid chromatography (HPLC) (Fig. 2D and E and Table S3). DPE mice had elevated levels of total dopamine analyte per unit protein (*P* = 0.011, Fig. 2D) and two out of three dopamine metabolites [3,4-dihydroxyphenylacetate (DOPAC), *P* = 0.38; homovanillic acid (HVA), *P* = 0.0063; 3-methoxytyramine (3-MT), *P* = 0.019; Fig. 2E]. Cysteinyl adducts cys-dopamine and cys-DOPAC were only detected above threshold in a subset of samples, with no group differences in the proportion of samples above threshold (cys-dopamine, *χ*^2^ test, *P* = 0.13; cys-DOPAC, *χ*^2^ test, *P* = 0.28). Cys-l-DOPA was detected in all samples but there were no differences between groups (*P* = 0.31).

### Vesicular dopamine uptake was unchanged in DPE mice

We assessed total dopamine uptake into striatal vesicles using a vesicular loading assay (Fig. S1 and Table S3). There were no differences between groups in total uptake per unit protein (*t* test, *N* = 12 per group, *t* = −0.79, *P* = 0.44).

### DPE altered electrophysiological properties of striatal medium spiny neurons

**Fig. 3.**
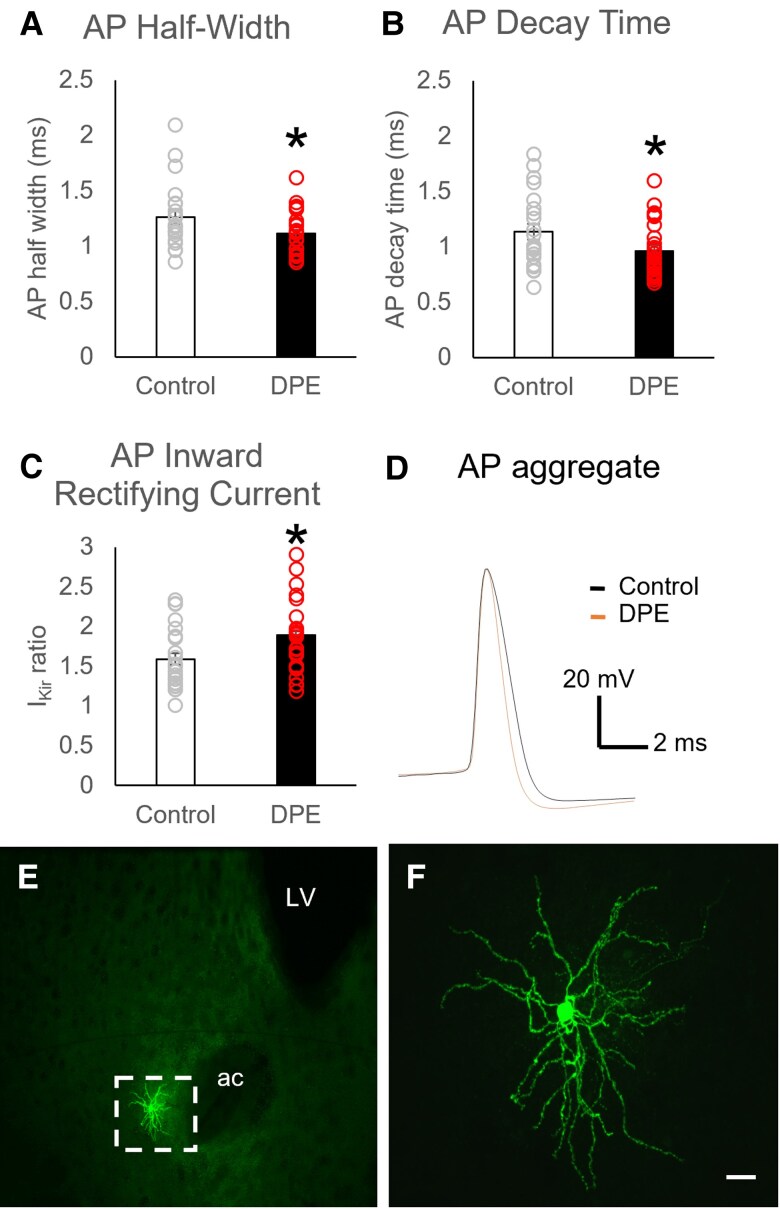
Changes in ventral striatum MSN properties in DPE mice. A) Action potential duration in MSNs, as measured by half-width, was reduced in DPE neurons (*N* = 22 neurons from 7 mice) relative to controls (*N* = 26 neurons from 6 mice), an effect driven by B) a faster action potential decay time. C) Inward-rectifying current was increased in MSNs of DPE neurons. D) Aggregate representation of MSN action potentials. E, F) Representative images show E) the morphology of a medium spiny neuron and F) the location of one recording site (site 4) in the ventral striatum. Scale bar is 20 *µ*m. Error bars are SEM. *P < 0.05 by *t* test. ac, anterior commissure; LV, lateral ventricle.

We measured electrophysiological properties of medium spiny neurons (MSNs) in the ventral striatum via whole-cell patch clamp recordings (Fig. 3A–F and Table S3). There was a main effect of DPE on cellular properties [MANOVA, main effect of exposure, *F*(1,13) = 2.2, *P* = 0.037]. While action potential (AP) threshold and amplitude remained the same between groups (*P* > 0.1), action potentials in neurons from DPE mice had shorter durations [MANOVA, exposure × AP half-width, *F*(1,44) = 6.0, *P* = 0.018; Fig. 3A], primarily driven by a faster decay [MANOVA, exposure × AP decay time, *F*(1,44) = 5.8, *P* = 0.020; Fig. 3B] with a trend toward a faster rise [MANOVA, exposure × AP rise time, *F*(1,44) = 3.07, *P* = 0.087]. Nucleus accumbens core (NAc) neurons from DPE mice had a more pronounced inward rectifier current than those from control mice [MANOVA, exposure × *I*_Kir_ ratio, *F*(1,44) = 4.7, *P* = 0.035; Fig. 3C], but no difference in resting membrane potential (*P* > 0.1), suggesting a possible decrease in neuronal excitability.

## Discussion

The critical question these experiments were designed to answer was whether developmental exposure to the pyrethroid pesticide, deltamethrin, which was identified as a risk factor for NDD when pregnant women are exposed (23, 32), is a direct cause of behavioral phenotype relevant to NDD in mice. Our behavioral testing strategy was to assay several domains of murine behaviors relevant to NDD, to include multiple behavioral tests in each domain, to include tests across the lifespan, and to use multiple independent cohorts to validate the findings. In both the discovery and replication cohorts, DPE mice showed reduced USVs, increased repetitive grooming, increased marble burying, decreased fear recall, and decreased acquisition of operant conditioning. Deficits were detected across the lifespan, from postnatal days 6 and 7 (USVs) to adulthood. In a previous study (27), DPE mice exhibited ADHD-relevant behaviors, including hyperactivity, impulsive responding, and repetitive arm entries in a Y-maze assay. The collective behavioral phenotype depicted in these two studies is broadly relevant to several NDDs, including the core symptoms of ADHD, intellectual disability, and two of the classic core symptom domains in autism (communication, and restrictive and repetitive behaviors). In combination with human epidemiology studies, these findings suggest that low-dose developmental exposure to deltamethrin may be a causal factor that increases the likelihood of diagnosis of NDD, including autism, by causing or worsening some core symptoms and comorbidities.

The primary strategy we used for behavior testing was to use behavioral batteries across the lifespan. There are some concerns with this approach, in particular the possibility that exposure to earlier assays may influence behavior in later assays. We sought to minimize these effects by organizing behavioral assays from least to most stressful; by testing the most stressful assays (fear conditioning and operant conditioning) in separate animals; by replicating every finding in an independent cohort; and by using separate animals for biological assays; all of which are the gold standard approaches (2). Nonetheless, follow-up studies testing animals on only a single assay would improve confidence in our findings.

DPE mice showed multiple distinct repetitive behaviors in the marble burying and observation assays, which reduces concern over measure-specific confounds. Marble burying alone as an outcome measure is not without controversy. Marble-burying behavior is common in mouse models of autism, is correlated with repetitive digging, and may serve as a proxy for repetitive behavior in general (8, 53, 54). However, the assay's relevance to obsessive–compulsive disorder has been criticized for lack of predictive validity (55), and its relevance to anxiety-related behavior has been disproven (53). Nonetheless, DPE mice also showed increased self-grooming, and previous findings of increased same-arm entries in the Y-maze in DPE mice may also represent repetitive circling or restrictive behavior (56), although it is often framed as representing a working memory deficit (7).

In the contingency degradation task with the discovery cohort, DPE animals failed more frequently at the initial operant training phase, ultimately invalidating the results from contingency degradation. This finding prompted the inclusion of operant conditioning in the replication cohort. Similarly, fear-conditioned DPE siblings used as stimulus animals for the social fear transmission task showed a decrease in freezing during fear recall, prompting the inclusion of classic fear conditioning in the replication cohort. The complementary deficits observed in these two very different tasks reduce concerns over potential task-specific confounds. Fear conditioning and operant conditioning classically rely on very different brain mechanisms, suggesting broader neurological changes than the striatum-specific effects described here, but one potential overlap is that both behaviors rely on dopamine released from dopamine neurons in the ventral tegmental area, a critical part of the mesolimbic dopamine pathway (57).

Importantly, we did not detect any differences in the social behavior domain in DPE mice in juvenile social interaction, adult social interaction, social approach, or social fear transmission. Several of the most common mouse models of autism also lack social deficits, including most genetic knockouts of neuroligin, neurexin, and Shank1 (7). However, the goal of these experiments was not to demonstrate that DPE mice are a model of autism, which is an outdated formulation (2, 58). Instead, we sought to determine whether the behavioral and molecular phenotype in mouse that is caused by the developmental exposure is relevant to autism and NDD in general, suggesting that the developmental exposure in humans may be a direct cause in addition to being a risk factor.

A major strength of this study is the use of discovery and replication cohorts, as well as multiple measures of each phenotypical element, which dramatically increases confidence that the observed differences are real. Additionally, different cohorts were tested in different facilities at Emory University and used mice from different origins due to facility restrictions (cohort A in Whitehead, using C57BL6/CR, and cohort B and replication cohort at Yerkes, using C57BL6/J), demonstrating the robustness of the replicated results and the generalizability across strains and testing conditions.

Elevated levels of dopamine and dopamine metabolites were detected in the dorsal and ventral striata of DPE mice, along with an increase in stimulated dopamine release. One critical question for determination of downstream molecular effects and treatment strategies is which compartment contains this excess dopamine. Previous reports showed a decrease in extracellular dopamine in the striatum of DPE mice, potentially caused by an increase in striatal DAT (27). Total vesicular uptake of dopamine in striatal samples did not differ between control and DPE mice, suggesting that the total dopamine content of vesicles was unaffected by DPE. Dopamine that is transported out of the extracellular space but not sequestered in vesicles is most likely to be free-floating in the cytosol, and excess cytosolic dopamine is classically associated with oxidative stress (59, 60). However, no differences were detected in cysteinyl adducts of dopamine, which are common indicators of dopamine auto-oxidation.

Elevation of the dopamine metabolite HVA in blood, urine, or cerebrospinal fluid (CSF) was one of the earliest potential biomarkers associated with autism and is consistently identified in metabolomics studies of autistic individuals (61–66). Here, we observed that DPE mice also have increased levels of the dopamine metabolites HVA and 3-MT in the striatum. Degradation of dopamine into its metabolites occurs through the sequential action of two enzymes, catechol-*O*-methyltransferase (COMT) and monoamine oxidase (MAO), which modify different motifs on the dopamine molecule (60). When dopamine degradation is initiated by COMT, the by-product is 3-MT; when degradation is initiated by MAO, the by-product is DOPAC; and both metabolites are further degraded to HVA. Interestingly, COMT is primarily expressed in glial cells and mostly absent from neurons (60, 67). The presence of elevated 3-MT, but not DOPAC, may suggest that glial degradation of dopamine predominates in DPE mice. Astroglia play a major role in the containment and degradation of reactive oxygen species, and it may be the case that elevated DAT on synapse-adjacent astroglia serve to move a significant portion of the excess dopamine into the glial cytosol for degradation into 3-MT and HVA. An astroglia-specific alteration on dopamine degradation could also explain the lack of cysteinyl adducts. Astroglia are important for the central nervous system's response to environmental insults and may play a role in the etiology of autism (68).

We also found that postsynaptic MSNs in the striatum of DPE mice had altered electrophysiological properties. Striatal MSNs of DPE mice showed shorter action potential durations (primarily owing to a faster decay time), with normal action potential threshold and amplitude. Action potential repolarization is primarily driven by potassium conductance, with a fast decay suggesting an increased conductance. Consistently, these neurons in DPE mice also showed an increased inward-rectifying potassium current (*I*_Kir_), which plays a key role in both action potential duration and neuronal excitability. There is evidence associating gain-of-function channelopathy in at least two subtypes of inward-rectifying potassium channels (Kir2.1 and Kir4.1) with autism, and Kir2.1 is highly expressed in striatal neurons, making this an interesting target for future investigation (69–72). Overall, these findings suggest that striatal MSNs in DPE mice have decreased neuronal excitability.

Many neurons exhibit activity-dependent homeostasis, in which neuronal excitability is broadly reduced in response to excess excitatory input. In striatal neurons in particular, potassium channels are recruited to promote homeostatic regulation and limit intrinsic neuronal excitability (73–75). Our observed reduction in striatal MSN excitability may represent an adaptation to excess dopamine release, in which one or more types of potassium channels are recruited to increase inward current. One paradoxical effect of this homeostatic mechanism would be to increase the relative contribution of dopamine to overall striatal MSN firing, potentially contributing to dopamine-related behavioral phenotypes such as hyperactivity, attention, repetitive behaviors, and learning and memory.

Together, these findings support the following conclusions about dopamine in DPE mice: the excess in striatal dopamine is neither extracellular nor sequestered in vesicles; the increase in dopamine release is counteracted by an increase in dopamine transporters moving dopamine out of the extracellular space; the excess dopamine is likely to be in the presynaptic cytosol, or the astroglial cytosol, or both; and striatal MSNs adapt by reducing overall excitability.

A major limitation of this study is the reliance on a single exposure dose of deltamethrin. This dose was selected based on the developmental “lowest observable adverse effect level” from prior studies using this model (27, 76) and is well below both the lower limit of the benchmark dose used for regulatory guidance (10.1 mg/kg) and the developmental “no observable adverse effect level” from prior literature (12 mg/kg) (76). While the exposure-dependent levels of deltamethrin and its metabolites in the blood and tissues of pregnant mice and humans have not been measured, findings from other studies offer some context. Exposure to approximately 1 mg/kg in adult rat led to deltamethrin levels in blood of 56 ng/mL and brain of 12 ng/g (77). The predicted accumulation in human from the same 1 mg/kg dose using physiologically based models is approximately twice this concentration, suggesting an increased sensitivity (78). By comparison, pregnant mice exposed to 1.5 mg/kg permethrin showed serum concentrations of 260 ng/mL (79). A study on deltamethrin levels in blood in the general population in Beijing showed a range of 0–17.34 ng/mL (80), and a separate study in pregnant Chinese women showed an average level in blood of 39 ng/mL (81). Urinary deltamethrin metabolite levels have also risen progressively in the US general population, from an average of 0.292 ng/mL in 1999–2000 (82), to 410 ng/mL in 2007, and to 660 ng/mL in 2011–2012 (83).

Another limitation of this study is the focus on male subjects in most assays. Prior studies of DPE in mice showed behavioral and molecular effects almost exclusively in males (27, 76). Additionally, in these experiments, we measured USVs in both male and female offspring and saw a reduction in vocalizations only in DPE males. These findings strongly suggest a primary sex-dependent effect of DPE. Nonetheless, the absence of additional data in DPE females represents a lost opportunity to more fully describe sex differences in response to DPE. Another lost opportunity was the lack of inclusion of behavioral tests addressing the important domains of sensory and motor behaviors, which can sometimes underly more complex behavioral deficits.

In summary, mice developmentally exposed to a low dose of deltamethrin show a behavioral phenotype relevant to autism, ADHD, intellectual disability, and NDD in general. DPE mice also have multifaceted disruptions in the dopamine system, including increased striatal dopamine content, release, reuptake, and metabolism, along with compensatory changes in MSN electrical properties. Several molecular signatures observed in DPE mice have direct synergy with molecular signatures observed in autistic patients, suggesting that this exposure model may provide translational value. Consideration of the evidence from our animal model corroborates the link between pyrethroids and both autism and developmental delay from the CHARGE study (23) and supports the interpretation that low-dose developmental pyrethroid pesticide exposure is a causal factor that increases the likelihood of diagnosis of NDD. A reconsideration of the EPA's benchmark dose for deltamethrin, used for regulatory guidance for all pyrethroid pesticides, is warranted.

## Materials and methods

### Animals

The experimental subjects used for our study were healthy adult (P56+) male and female offspring of female C57BL6/J or C57BL6/CR mouse dams. Virgin dams were kept on a 12/12 light cycle and given access to water and high-fat breeder diet (5015, LabDiet, St. Louis, MO) ad libitum. Dams were housed in female pairs, then in trios with a male sire until 3 days prior to birth, and then singly housed through birth and weaning. Offspring were weaned at P20–22 into same-sex cages and received water and standard mouse diet (5001, LabDiet, St. Louis, MO) ad libitum. A subset of offspring was weaned into mixed litter same-sex cages for use in operant conditioning. All procedures were approved by the Emory University IACUC and performed in accordance with the US Animal Welfare Act.

### Study design

The experimental design consisted of two groups: developmental deltamethrin exposure (DPE) and control. Female mouse dams were exposed to deltamethrin (or control) during preconception, pregnancy, and lactation as described below (Fig. 1A). The dam's offspring were the subjects of the subsequent experiments.

### Experimental units

Since experimental treatment was given to the dam, the experimental unit was the litter, with each litter counted as *N* = 1. In all experiments, each test subject was a representative animal chosen from its litter, so the *N* for each experiment represents both the number of animals and the number of litters. Patch clamp was the exception; the experimental unit was the neuron, with 2–3 neurons per animal recorded from a representative animal from its litter. To minimize cross-interference between tests, different animals from the same litter were used for behavior tests involving foot shocks (fear conditioning and social fear), food restriction (contingency degradation and operant conditioning), and tissue collection; no animal experienced more than one of these. Because the primary effects of DPE on learning outcomes and molecular outcomes were previously observed in males (27), most behavior tests and all molecular assays were conducted on males only. For separation-induced USVs, one male and one female pups from each litter were used, and sex was considered a within-subject factor.

### Cohorts

For behavioral experiments, a discovery cohort was used to select primary outcome measures of interest, which were compared between groups in a replication cohort. Experimental subjects were bred and tested in three cohorts. Discovery cohort A consisted of the offspring of female C57BL6/CR mouse dams (Charles River, Wilmington, MA) tested in the Emory University Whitehead animal facility. Discovery cohort B and the replication cohort consisted of the offspring of female C57BL6/J mouse dams (Jackson Labs, Bar Harbor, ME) tested in the Emory University Yerkes National Primate Center animal facility. Colony housing conditions and care were otherwise identical among cohorts.

### Sample size and elimination criteria

Dams that did not consistently consume the deltamethrin (as described below), or that did not give birth within 28 days of being housed with a sire, were eliminated a priori. In discovery cohort A, 12 females were offered doses of deltamethrin as described below. Two females were eliminated for insufficient consumption and one female did not produce a litter within the specified time frame, leaving nine litters (control, *N* = 4; DPE, *N* = 5). In discovery cohort B, 20 females were offered doses; two were eliminated for insufficient consumption and two did not produce a litter, leaving 16 litters (control, *N* = 7; DPE, *N* = 9). In the replication cohort, 40 females were offered doses; three were eliminated for insufficient consumption and two did not produce a litter, leaving 35 litters (control *N* = 16; DPE *N* = 19). One litter in the replication cohort contained only a single male, and another litter contained only two males; these males were divided between operant conditioning (control *N* = 16; DPE *N* = 19) and fear conditioning (control *N* = 15; DPE *N* = 19), leaving 33 litters for all other tests (control *N* = 14; DPE *N* = 19). There were no differences between groups in eliminations.

### Blinding

Offspring cages were assigned numbers randomly at weaning, and all subsequent tests were performed using these numbers. Experimenters remained blind to the treatment until after data analysis was concluded.

### Statistics

The analysis strategy for behavioral experiments was to use discovery cohorts to select primary outcome measures of interest and a directional hypothesis for each measure, which were then tested using an independent replication cohort. When possible, one-tailed statistical tests were used in the replication cohort because the only outcome of scientific interest was whether the effect from the discovery cohort was replicated. Cohorts were then combined using ANCOVAs, with cohort as a covariate, for the determination of combined *P*-value and effect size. All statistics were calculated as described using SPSS version 27 (IBM, Armonk, New York) with a significance threshold of *α* = 0.05. Parametric data points greater than 3 SD from the mean were defined a priori as outliers, but no outliers were identified in any of our data.

### Chemicals

Deltamethrin (>98% pure, Sigma, St. Louis, MO), the reference pyrethroid selected by the EPA (24), was dissolved in acetone (Sigma) and mixed with corn oil (Mazola, Memphis, TN). The acetone was then allowed to evaporate overnight. The resulting colloid was aliquoted into borosilicate glass vials to avoid seepage into plastic containers and stored at −80°C until the day of use. Aliquots of vehicle were prepared by allowing a similar volume of acetone to evaporate in corn oil. Commercial peanut butter (JIF, J.M. Smucker, Orville, OH) and corn oil were not analyzed for pre-existing pesticide content.

### Developmental DPE

Mice were developmentally exposed to the pyrethroid pesticide deltamethrin as described previously (27). Starting 2 weeks prior to being paired with a male, dams were fed deltamethrin (3 mg/kg orally in peanut butter/corn oil) or vehicle once every 3 days, continuing throughout preconception, pregnancy, and the lactation period. Time of day was not strictly controlled but all feedings occurred between 9 AM and 5 PM. Dams were weighed immediately prior to each feeding, and the appropriate amount of corn oil (1 *µ*L/3 g mouse, ∼6–10 *µ*L average) was mixed with JIF peanut butter (∼100 mg; J.M. Smucker, Orville, OH) to achieve the desired dose. Experimenters were blind to the contents of the corn oil mixture during exposures. The mixture was transferred onto the wire food hopper such that the dam could voluntarily consume it without additional handling stress and in a position where offspring would not have access. Home cage barriers were used during feedings to ensure individual dosing in the presence of adult cagemates. Dams that failed to consume 90% of the peanut butter during 90% of the feedings were eliminated from the study. Offspring were only exposed to deltamethrin indirectly through the dam. Offspring were weaned from the dam as above and received no additional exposure.

### Behavioral batteries

Multiple complementary behavioral assays were selected to address each of four multiple domains relevant to autism and NDD across the lifespan: social behavior, restrictive and repetitive behaviors, communication, and cognition (Tables S1 and S2). To minimize repeated testing stress as a confounder, assays were organized into fixed-order behavioral batteries in order of increasing stress, with the least stressful assays performed first. Assays involving foot shock (i.e. fear conditioning and social fear) were performed on separate mice from the same litters to ensure no overlap. Similarly, assays involving food restriction (i.e. operant conditioning and contingency degradation) were performed on separate mice from the same litters, and these mice were not challenged with any other behavioral assays. Finally, separate behavior-naïve mice from the same litters were used for all tissue collection and biological assays. Male mice only were used for all assays except separation-induced USVs.

Offspring in the discovery cohorts were challenged with one of three different behavioral batteries: (i) contingency degradation only (with operant conditioning phase); (ii) a fixed-order behavioral battery consisting of (a) separation-induced USVs, (b) maternal potentiation of USVs, (c) juvenile play, (d) marble burying, (e) three-chamber social approach, (f) social interaction (with a novel cage observation phase), and (g) classic fear conditioning; or (iii) the same battery as in (ii) but with social fear conditioning instead of classic fear conditioning. We selected primary outcome measures of interest based on these experiments in the discovery cohort and challenged the offspring in the replication cohort with either (i) operant conditioning or (ii) a fixed-order behavioral battery consisting of (a) separation-induced USVs, (b) marble burying, (c) novel cage observation, and (d) classic fear conditioning. Methods for the behavioral assays conducted on the discovery cohorts appear in the Supplementary Methods, and results for these assays are summarized in Table S1. Methods and results for the behavioral assays conducted on the replication cohort appear below and are summarized in Table S2.

### Separation-induced USVs

Separation-induced USVs were observed at postnatal day 6 and 7 in one male pup and one female pup per litter from the replication cohort. Two litters were eliminated when their sessions failed to record, resulting in data from *N* = 14 control litters and *N* = 17 DPE litters. The sex of pups was determined by the presence or absence of a black spot on the abdomen of males and/or inguinal teats in females. The order of testing was counterbalanced across sex. After the home cage was moved to the experimental room, one pup was transferred to a testing cup that was then placed in a soundproof recording chamber (IAC Acoustics, North Aurora, IL). Recordings were taken using a one-fourth-inch microphone attached to a conditioning amplifier (Brüel and Kjær, Nærum, Denmark) and controlled with MATLAB software (MathWorks, Natick, MA). USVs were recorded for 5 min. The second pup from the same litter was tested in the same manner.

Call number was selected as the primary measure of interest and was compared between groups using a 2×2 ANOVA, with exposure (control or DPE) as a between-subject factor and sex (male or female) as a within-subject factor. Post hoc one-tailed *t* tests were used to compare exposures within each sex.

### Novel cage observation

Adult male subjects in the replication cohort (control, *N* = 14 litters; DPE, *N* = 19 litters) were placed alone in an empty cage and digitally recorded for 10 min. Recordings were scored manually using The Observer software (The Observer 10.0, Noldus, Wageningen, The Netherlands), with self-grooming as the primary outcome of interest. Self-grooming was compared between groups using a one-tailed *t* test.

### Marble burying

Adult male subjects in the replication cohort (control *N* = 14 litters, DPE *N* = 19 litters) were tested for marble-burying behavior as previously described (84). Briefly, each subject was placed in a clean cage with 2 inches of bedding and 20 marbles. After 30 min, the mouse was removed and the testing cage was photographed. The number of buried marbles was determined by counting the number of visible marbles less than two-thirds obscured by bedding and subtracting from 20. The number of marbles buried was compared between groups using a one-tailed *t* test.

### Classical fear conditioning

The fear conditioning protocol was optimized to detect a decrease in cued fear recall in the experimental group. Immediately prior to the test, one subject was eliminated for fighting. On day 1, the remaining adult mice in the replication cohort (control, *N* = 15 litters; DPE, *N* = 18 litters) were each placed alone in a conditioning chamber (San Diego Instruments, San Diego, CA). Their activity was recorded for 7.5 min in context A as they were exposed to a fear conditioning protocol consisting of a 3-min habituation period followed by five presentations of a tone (30 s, 6 kHz, 90 dB) co-terminating with a mild foot shock (0.3 mA, 0.5 s) with 30-s intertrial intervals. On day 2, mice were returned to the conditioning chamber under context B and were exposed to an identical protocol without foot shocks. The primary outcome measure was freezing behavior during tone presentations on day 2. Descriptive data on baseline freezing and within-session fear acquisition from day 1 were collected and analyzed.

Cued fear recall was chosen as the primary outcome of interest. Data in the replication cohort were parametric and not transformed for analysis. Average freezing during tones in the fear recall test was analyzed using a one-tailed *t* test. Descriptive measures of baseline freezing and within-session fear acquisition were also analyzed.

### Fixed ratio operant response training

Adult male mice in the replication cohort (control, *N* = 16; DPE, *N* = 19) were trained to nose poke for food as previously described (85, 86). Briefly, mice were food restricted to maintain around 90% of their free-feeding weight. Each day, mice were placed in an operant conditioning chamber (Med Associates, At. Albans, VT) and given access to two nose poke apertures and a food magazine. Mice could receive up to 30 food reinforcements (20 mg Bio-Serv pellets, Bio-Serv, Flemington, NJ) from each aperture on a FR1 schedule, for a total of up to 60 reinforcements per day. Each daily FR1 training session for each mouse continued until all 60 reinforcements were earned or for a maximum of 70 min. Daily training sessions were ended after exactly 7 days to give all mice the same amount of time to pass the acquisition criterion of 30 reinforcements earned on each aperture.

Acquisition criterion was chosen as the primary outcome of interest. A one-tailed *χ*^2^ table test was used to compare acquisition criterion pass/fail ratios between groups following FR1 training. Descriptive 7-day acquisition curves were constructed and analyzed using nose poke rate data.

### Dissections

A randomly selected subset of behaviorally naïve male mice was sacrificed at adulthood via rapid decapitation for tissue collection for HPLC (control *N* = 7, DPE *N* = 10) and vesicular loading assay (discovery: control *N* = 3, DPE = 5; replication: control *N* = 12, DPE *N* = 12). Animals were rapidly decapitated, brains were dissected and placed on a chilled aluminum block (Thermo Scientific, Waltham, MA), and the striatum (dorsal and ventral) was isolated using razor blades. Striatal samples were placed in Eppendorf tubes, flash frozen in liquid nitrogen, and stored at −80°C until needed.

### Fast-scan cyclic voltammetry

Dopamine release and reuptake kinetics were quantified using fast-scan cyclic voltammetry as previously described (84, 87). Randomly selected, behaviorally naïve adult male mice (control *N* = 6, DPE *N* = 8) were rapidly decapitated, and striatal sections were immediately collected via vibratome as previously described. For dorsal striatum, dopamine release was evoked by a single, rectangular, electrical pulse (300 *μ*A, 2 ms per phase, biphasic); for ventral striatum, five pulses were delivered at 60 Hz. Five recordings were taken at each of three sites in the dorsal striatum and three sites in the ventral striatum, with a 5-min rest interval between each stimulation. Electrodes were calibrated to known dopamine standards to produce estimated dopamine concentrations. Maximal dopamine release at dorsal and striatal sites were averaged separately and compared using a two-way ANOVA, with region as a within-subject factor and exposure as a between-subject factor.

### HPLC

The Emory University HPLC Core performed HPLC on dissected striatal samples from adult male mice (control *N* = 7, DPE *N* = 9) to measure dopamine, its metabolites, and its cysteinyl adducts as described previously (88). Monoamines, including dopamine, DOPAC, HVA, and 3-MT, and cysteinyl analytes, including cys-dopamine, cys-DOPAC, and cys-L-DOPA, were examined by HPLC with electrochemical detection as described previously (89). The analytes were identified by the matching criteria of retention time and sensor ratio measures to known standards (Sigma, St. Louis, MO). Compounds were quantified by comparing peak areas to those of standards on the dominant sensor.

The concentration of analyte per unit mass of tissue in the sample was calculated by dividing the concentration of analytes as determined by HPLC by the total protein concentration from the bicinchoninic acid (BCA) assay. These concentrations (in ng analyte/mg protein) were compared between groups using *t* tests.

### Vesicular loading assay

Vesicular dopamine uptake was measured using dissected striatal samples from adult male mice (discovery: control *N* = 3, DPE = 5; replication: control *N* = 12, DPE *N* = 12) as previously described (90). Counts were normalized to a [^3^H] radioactivity standard and to total protein, as determined by BCA assay. Differences between groups in each cohort were assessed using *t* tests.

### Slice electrophysiology

Whole-cell patch clamp electrophysiology was used to measure properties of ventral striatal MSNs. Adult male mice (6 control, 7 DPE, 2–3 neurons per subject, for a total of *N* = 22 control neurons and *N* = 26 DPE neurons) were used for electrophysiology experiments. Brain slices containing the NAc were prepared using a previously described method (91) with slight modifications. Briefly, mice were decapitated under deep isoflurane anesthesia (Henry Schein Inc., Melville, NY, USA), and the brain was quickly removed from the skull and immersed in ice-cold (4°C) 95–5% O_2_/CO_2_ oxygenated “cutting solution” with the following components: NaCl (130 mM), NaHCO_3_ (30 mM), KCl (3.50 mM), KH_2_PO_4_ (1.10 mM), MgCl_2_ (6.0 mM), CaCl_2_ (1.0 mM), glucose (10 mM), and kynurenic acid (2.0 mM). Coronal sections with thickness of 300 *µ*m containing the nucleus accumbens were cut using a vibratome (Leica VTS-1000 Leica Microsystems, Bannockburn, IL, USA) and were kept in oxygenated cutting solution in an incubation chamber at 32°C for 1 h. Slices were then transferred to regular artificial cerebrospinal fluid (ACSF) containing the following components: NaCl (130 mM), NaHCO_3_ (30 mM), KCl (3.50 mM), KH_2_PO_4_ (1.10 mM), MgCl_2_ (1.30 mM), CaCl_2_ (2.50 mM), and glucose (10 mM). Slices were kept in ACSF at room temperature for at least 30 min before recording.

For whole-cell patch clamp recording, slices were moved to the recording chamber and continuously perfused by gravity-fed oxygenated ACSF at 32°C (∼2 mL/min). Individual neurons in the NAc were visualized using differential interference contrast optics and infrared (IR) illumination with an IR-sensitive charge-coupled device (CCD) camera (Orca ER, Hamamatsu, Tokyo, Japan) mounted on a Leica DM6000FS microscope (Leica Microsystems Inc., Bannockburn, IL). Patch pipettes were pulled from borosilicate glass with resistance between 4 and 6 MΩ and were filled with patch solution containing the following: K-gluconate (130 mM), KCl (2 mM), HEPES (10 mM), MgCl_2_ (3 mM), phosphocreatine (5 mM), K-ATP (2 mM), and 0NaGTP (0.2 mM) with pH adjusted to 7.3 with KOH and an osmolarity of 280–290 mOsm. Whole-cell recordings were performed with a Multiclamp 700B amplifier (Molecular Devices Corporation, Sunnyvale, CA) using pClamp 10.4 software and an Axon Digidata 1550 A-D interface (Molecular Devices Corporation). NAc MSNs were visually identified by their medium soma size and confirmed by their membrane properties (92). Whole-cell access resistances were in the range of 5–20 MΩ and were routinely monitored during the experiment; a change of <15% was deemed acceptable. The membrane potential was held at −70 mV for all neurons, and only those neurons with a stable membrane potential more negative than −60 mV were used. Intrinsic membrane currents and action potential firing pattern were examined under current-clamp mode with a 750-ms current step injection protocol, as previously described (91). A linear ramp protocol (250 ms) was used to create a single action potential for analysis of action potential kinetics. Spontaneous excitatory postsynaptic currents (EPSCs) were isolated in the presence of the GABA_a_ antagonist SR 95531 (5 *µ*M) and were captured continuously for 2 min, and EPSC events were detected offline using the MiniAnalysis program 6.0 (Synaptosoft Inc., Decatur, GA). *t* tests were used to compare results between exposure groups. The experimenter remained blind to the identity of all mice until all analyses were completed.

## Supplementary Material

pgad085_Supplementary_Data

## Data Availability

The authors confirm that all raw and processed data supporting the findings in this study are available within the article and its associated supplementary materials and data sets.
