# Supplementary material for: Developmental pyrethroid exposure causes a neurodevelopmental disorder phenotype in mice
Source: PNAS Nexus. 2023 Apr 25;2(4):pgad085. doi: 10.1093/pnasnexus/pgad085 (PMC10129348; doi:10.1093/pnasnexus/pgad085)
Supplement: pgad085_Supplementary_Data [file pgad085_supplementary_data.zip › PNASNEXUS-PNASNEXUS-2022-00680-s01.docx]

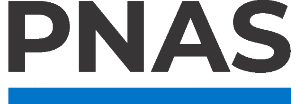


**Supplementary Information for**

Developmental pyrethroid exposure causes an autism-related phenotype in mice.

Melissa A. Curtis^1^, Rohan K. Dhamsania^2,3^, Rachel C. Branco^4,5^, Ji-Dong Guo^6^, Justin Creeden^7^, Kari L. Neifer^1^, Carlie A. Black^4,8^, Emily J. Winokur^2,9^, Elissar Andari^6,10^, Brian G. Dias^6,11^, Robert C. Liu^12^, Shannon L. Gourley^13^, Gary W. Miller^14,15^, James P. Burkett^1,14*^

^1^ Department of Neurosciences, University of Toledo College of Medicine and Life Sciences, Toledo, OH 43614 (current)

^2^ College of Arts and Sciences, Emory University, Atlanta, GA 30322

^3^ Philadelphia College of Osteopathic Medicine, Philadelphia, PA 19131 (current)

^4^ Laney Graduate School, Emory University, Atlanta, GA 30322

^5^ Department of Chemistry and Biochemistry, University of Notre Dame, Notre Dame, IN, 46556 (current)

^6^ Department of Psychiatry and Behavioral Sciences, Emory University School of Medicine, Atlanta, Georgia 30322

^7^ Department of Medicine, University of Toledo College of Medicine and Life Sciences, Toledo, OH 43614

^8^ Schiemer School of Psychology and Biblical Counseling, Truett McConnell University, Cleveland, GA 30528 (current)

^9^ Department of Cognitive Science, University of California San Diego, La Jolla, CA 92093 (current)

^10^ Department of Psychiatry, University of Toledo College of Medicine and Life Sciences, Toledo, OH 43614 (current)

^11^ Department of Pediatrics, Keck School of Medicine of USC, Los Angeles, CA; Division of Endocrinology, Children’s Hospital Los Angeles, Los Angeles, CA; Developmental Neuroscience and Neurogenetics Program, The Saban Research Institute, Los Angeles, CA (current)

^12^ Department of Biology, Emory University, Atlanta, Georgia 30322; Center for Translational Social Neuroscience, Emory University, Atlanta, Georgia 30322

^13^ Department of Pediatrics, Children’s Healthcare of Atlanta, Emory University School of Medicine, Atlanta, Georgia 30322; Emory National Primate Research Center, Atlanta, Georgia, 30329

^14^ Department of Environmental Health, Emory Rollins School of Public Health, Atlanta, GA 30322

^15^ Department of Environmental Health Sciences, Mailman School of Public Health, Columbia University, New York, NY 10032

*Corresponding author:
James P. Burkett
3000 Arlington Ave
Block Health Sciences #185
Toledo, OH 43614
419-383-4203
james.burkett@utoledo.edu

**This PDF file includes:**

Supplementary Methods

Supplementary Results

Supplementary References

Tables S1 to S3

Figure S1

**Supplementary Methods:**

*Discovery cohort.* Male and female offspring in the discovery cohorts were exposed to a fixed-order behavioral battery consisting of separation-induced USVs, maternal potentiation of USVs, juvenile social behavior, marble burying, three-chamber social approach, social interaction (with a novel cage observation phase), and one of either classic fear conditioning, social fear conditioning (with a classic fear conditioning phase) or contingency degradation (with an operant conditioning phase). Methods used in the discovery cohorts appear below.

*USVs.* At PND 6-7, one male and one female pup per litter (control N=3 litters, DPE N=5 litters) were tested for separation-induced USVs and maternal potentiation of USVs^1^. The sex of pups was determined by the presence of a black spot on the abdomen of males and/or inguinal teats in females. The order of testing was counter-balanced across sex. First, the home cage was moved to the experimental room and placed on a heating pad. The dam was isolated in a holding cage, and pups were left alone for 15-20 minutes prior to testing. One pup was then transferred to a testing cup that was placed in a soundproof recording chamber (IAC Acoustics, North Aurora, IL). Recordings were taken using a ¼” microphone attached to a conditioning amplifier (Brüel and Kjær, Nærum, Denmark) and controlled with Matlab software (MathWorks, Natick, MA). USVs were recorded for 5 minutes. The second pup from the same litter was tested in the same manner.

Immediately following the initial 5-minute recording session, pups were tested on maternal potentiation of USVs. Upon being removed from the soundproof chamber, each pup was placed into the holding cage with the dam for 5 minutes. After 5 minutes with the dam, the pup was returned to the recording chamber for an additional 5 minutes. The pup was then returned to the home cage. The second pup from the same litter was tested in the same manner.

Both recordings from each subject were analyzed using Matlab to identify the total number of calls and individual call features. Because the call number data were non-parametric, we transformed the data to ranks and normalized by dividing by the highest rank. Call number was compared between treatments using a 2x2x2 ANOVA, with exposure (control, DPE) as a between-subjects factor, and sex (male, female) and session (first, second) as within-subjects factors. Post-hoc t-tests compared group differences in each session. Call features were analyzed with a separate MANOVA, with factors of exposure and sex, and twenty call features as dependent measures.

*Juvenile social behavior.* At PND 20-22, immediately prior to weaning, male and female subjects (discovery: control N=4 litters, DPE N=5 litters) were tested for juvenile social behavior as previously published by McFarlane et al ^2^. Briefly, on the day before weaning, subjects were removed from the home cage, weighed, and marked with odorless colored marker. Subjects were placed in an empty cage for 1 hour and then transferred to a second empty cage for 30 minutes, before being returned to the home cage. On the day of weaning, offspring were placed in an empty cage for 1 hour and then transferred to a cage containing an unfamiliar, same-sex, age-matched, weight-matched social partner from the same treatment group for 30 minutes. One animal in the pair was designated as the subject and the other as the stimulus animal. This session was digitally recorded and later scored for social play, self-grooming, following, face sniffing, body/genital sniffing, allogrooming and huddling. No social play was observed by any subjects, so this measure was removed from analysis. All measures were compared between groups using t-tests.

*Marble burying.* Adult male subjects (control N=4 litters, DPE N=5 litters) were tested for marble-burying behavior as described in the Methods.

*Social approach.* After 8 weeks of age, adult male subjects (discovery: control N=4 litters, DPE N=5 litters) were tested for social approach using the procedure from McFarlane et al^2^. Briefly, subjects were placed in a three-chambered cage for four 10-minute sessions. In the first session, subjects habituated to the center chamber with barriers blocking the left and right. In the second session, subjects habituated to the full chamber with no barriers present. For the third session, subjects had full access to all chambers: one side contained an unfamiliar, same-sex, age-matched social partner confined underneath a wire pencil holder, and the other side contained an empty pencil holder. In the fourth session, subjects had full access to all chambers: one side contained the same partner from the previous session, and the other contained a new and unfamiliar (same-sex, age-matched) social partner. The second, third, and fourth sessions were digitally recorded and analyzed separately for time spent in proximity to each wire pencil holder and crosses in and out of each chamber. Time spent in proximity to the two wire cups during sessions 2, 3 and 4 was compared using three ANOVAs, with stimulus (session 2, left vs. right; session 3, social vs. object; session 4, familiar vs. unfamiliar) as a within-subjects factor and treatment (control vs. DPE) as a between-subjects factor. Data from session 2 were used to determine presence or absence of a side bias. Chamber crosses were compared between groups as a movement control.

*Social interaction.* On a new testing day following the social approach test, adult male subjects (discovery: control N=4 litters, DPE N=5 litters) were tested on social interaction using the procedure from McFarlane et al^2^. Briefly, male subjects and a designated unfamiliar, same-sex, age-matched, weight-matched stimulus animal were removed from the home cage and placed in clean, empty cages for 10 minutes. After 10 minutes, the stimulus animal was transferred into the cage of the subject for 10 minutes. Both 10-minute periods for the subject were digitally recorded, and the second session was later scored for self-grooming, following, face sniffing, body/genital sniffing, allogrooming and huddling. All measures were compared between groups using t-tests.

*Novel cage observation.* Using the procedure from McFarlane et al^2^, digital recordings from male subjects (control, N=4 litters; DPE, N=5 litters) from the 10-minute solo habituation phase of the social interaction test were scored for self-grooming, digging, inchworming and circling. No circling was observed so the measure was removed. Because the measures were non-parametric, we transformed the data to ranks and analyzed them using a MANOVA, with between-subjects factor of exposure and three dependent measures.

*Classical fear conditioning and social fear transmission.* Adult male mice in the discovery cohort were exposed to either classical fear conditioning or a social fear transmission protocol. Mice housed in cages of 2-3 same-sex siblings in the same treatment group were used as subjects. One sibling was designated as the “observer” (control, N=4 litters; DPE, N=5 litters), and another sibling was designated the “demonstrator” for the social fear transmission test. On Day 1 (fear acquisition), the demonstrator was removed from the home cage, placed alone in a conditioning chamber, and exposed to a 15.5 minute classic fear conditioning session, consisting of a 5-minute habituation period followed by five presentations of a tone (30 s, 6 kHz, 90 dB) co-terminating with a foot shock (0.8 mA, 0.5 s) at 2-minute inter-trial intervals. Training occurred in context A, which consisted of a darkened chamber wiped thoroughly with quatricide. On Day 2 (social fear transmission), the untrained observer was placed in the conditioning chamber in context A together with the demonstrator and allowed to freely interact while both mice were exposed to the same tone schedule as Day 1 without foot shocks. On Day 3 (fear recall), each sibling was placed alone in a conditioning chamber and again exposed to tones without foot shocks. Fear recall was tested in context B, in which the lights were on and the cage was wiped thoroughly with 70% ethanol. The primary outcome measure was freezing behavior, as measured during habituation and tone presentations on Day 1 (within-session fear acquisition) or Day 3 (cued fear recall).

In the discovery cohort, freezing data were non-parametric, so they were transformed to ranks and normalized by dividing by the highest rank. Within-session fear acquisition by demonstrators on Day 1 was assessed using a 2x5 ANOVA, with exposure (control, DPE) as a between-subjects factor and tone (5 tones) as a within-subjects factor. Post-hoc t-tests compared freezing between groups during each tone. Baseline freezing data collected during the habituation period was rank-transformed and analyzed separately with a t-test. Average freezing during tones in the fear recall session was analyzed separately in observers and demonstrators using t-tests. Freezing during habituation was also analyzed separately using t-tests.

*Operant conditioning experimental design.* Adult male mice were trained in an operant conditioning task in three phases. In the first phase, mice received 7-10 days of fixed response (FR) training, as described in the Methods. Mice that successfully passed the acquisition criterion went on to instrumental contingency degradation and random interval training, followed by three days of a response-outcome contingency degradation (CD) procedure, which tests the ability of subjects to associate actions with their outcomes. In the second phase, mice received four days of random interval (RI) training; this training elevates response rates and, with extensive training, induces habitual behavior that is insensitive to the outcome. This was followed by another three-day CD procedure. In the third phase, mice received another four days of RI training and a third CD procedure. These procedures are described in detail below.

*Fixed ratio operant response training.* Adult male mice in the discovery cohort (control, N=7 litters; DPE, N=9 litters) were trained to nose poke for food as described in the Methods, except that daily training sessions continued for each individual for a minimum of 7 days and up to 10 days until they reached the acquisition criterion of 30 reinforcements earned on each aperture. Data on nose poke rate on both apertures on the last 7 days of FR1 training for each mouse were used to construct the response acquisition curve. Three mice that never passed the acquisition criterion were excluded from further testing.

For the discovery cohort, a chi-squared table test was used to compare the failure to pass the acquisition criterion between groups following FR1 training. The FR1 acquisition curve (including all subjects) was analyzed using an ANOVA to compare nose poke rate between groups, with experimental day (1-7) as a within-subjects factor and treatment (control, DPE) as a between-subjects factor.

*Random interval operant response training.* After FR1 training, mice in the discovery cohort that passed the acquisition criterion (control N=7, DPE N=6) were exposed to two additional four-day random interval (RI) training phases, which used RI 30-second schedules. As with FR1 training, each session continued until 30 reinforcements per aperture (60 total) were earned, or for a maximum of 70 minutes. As before, the acquisition criterion was defined as earning 30 reinforcements in a single session on each aperture. One mouse failed to reach the acquisition criterion in the first RI training phase and was excluded from further testing. The full 15-day acquisition curve (including only subjects that passed the acquisition criterion in all three phases) was analyzed using a 2x15 ANOVA.

*Instrumental contingency degradation tests.* At the end of each of the three training phases described above (FR1, RI1 and RI2), mice in the discovery cohort (control N=6 litters, DPE N=6 litters) were tested with a contingency degradation procedure as previously described^3,4^. Briefly, mice underwent two days of CD training followed by a probe test on the third day. On the first day, mice were placed in an operant conditioning chamber in which one of the two previously available apertures was occluded, and responding on the available (or “non-degraded”) aperture was reinforced according to a RI 30-second schedule for 25 minutes. On the second day, the previously available aperture was occluded and the other aperture was available; however, food pellets were delivered non-contingently over 25 minutes at fixed intervals determined by the animal’s reinforcement rate from the previous day. This procedure “degrades” the predictive relationship between a given behavior (here, nose poke response) and its associated outcome. On the third day, both apertures were freely available during a brief 10-minute probe test, and no food pellets were delivered. Response rates at the “degraded” and “non-degraded” apertures over 10 minutes were recorded. A total of three CD tests were performed.

Nose pokes during each of the three contingency degradation tests were analyzed using a 3x2x2 ANOVA, with training epoch (1-3) and aperture (active, degraded) as within-subjects factors and treatment (control, DPE) as a between-subjects factor. Post-hoc paired t-tests compared responding on the active and degraded apertures within each epoch and each treatment group. Additional post-hoc t-tests compared aperture preference (active minus degraded nose pokes) between groups in each epoch.

**Supplemental Results:**

*USVs.* Male and female pups that were developmentally exposed to pyrethroid pesticide produced fewer distress vocalizations than controls across all sessions (ANOVA, main effect of treatment, F(1,6)=16.7, p=0.006; Table S1). Mice in all groups and of all sexes produced fewer vocalizations in the second session (ANOVA, main effect of session, F(1,6)=67.5, p<0.0005; post-hoc t-tests, p<0.05; Table S1), indicating that the maternal exposure protocol depresses USVs in mice rather than potentiating them. There was no effect of exposure on call features (MANOVA, no main effects or interaction effects; Table S1), indicating that the acoustic features of calls were unchanged. Therefore, call number was selected as the outcome measure of interest for the replication cohort.

*Juvenile social behavior.* There were no differences between treatment groups in either cohort on measures of self-grooming, following, face sniffing, body/genital sniffing, huddling or allogrooming (p>0.05, Table S1). Notably, there were insufficient instances of social play to make quantitative comparisons.

*Marble burying.* DPE mice buried significantly more marbles than control mice in a 30-minute session (t-test, t=-5.0, p=0.002; Table S1). Marbles buried was selected as an outcome measure of interest for the replication cohort.

*Social approach.* There was an overall preference for the social stimulus in the social approach phase (main effect of stimulus, F(1,7)=9.0, p=0.02) and a trend toward a novelty preference in the social memory phase (main effect of stimulus, F(1,7)=3.3, p=0.1), suggesting that all subjects showed social preference and social memory. However, there were no differences between treatment groups in the time spent in proximity to the social vs. non-social stimulus or in time spent in proximity to the familiar vs. unfamiliar stimulus (Table S1). There were no differences in total crosses in any session.

*Social interaction.* There were no differences between treatment groups on measures of self-grooming, following, face sniffing, or body/genital sniffing (p>0.05, Table S1). There were insufficient instances of allogrooming or huddling to make quantitative comparisons.

*Novel cage observation.* DPE mice performed more self-grooming than control mice during the 10-minute observation period (MANOVA, F(1,7)=10.8, p=0.013; Table S1). There were no differences on measures of digging or inchworming (p>0.1; Table S1). Self-grooming was chosen as the outcome measure of interest for the replication cohort.

*Social fear transmission.* In the discovery cohort, mice were exposed to classical fear conditioning and social fear transmission, and freezing during fear acquisition and fear expression was measured (Table S1). DPE stimulus mice (demonstrators) had a lower baseline of freezing than controls (t-test, t=-4.1, p=0.004; Table S1). During fear acquisition in demonstrators, both exposure groups acquired a within-session freezing response (ANOVA, main effect of tone, F(4,28)=19.1, p<0.0005). DPE demonstrator mice showed less freezing than controls during early tones (exposure x tone interaction, F(4,28)=5.2, p=0.003) but not overall (no main effect of exposure, F(1,7)=1.5, p=0.26). This reduced freezing at baseline and early in the conditioning session may reflect the general hypermobility of the DPE mouse^5^. During fear expression on Day 3, DPE demonstrator mice showed no difference in freezing during habituation (t-test, p=0.50) but less freezing during tones (t-test, p=0.039), suggesting a deficit in fear recall. Despite these differences in the fear response in demonstrators, DPE observer mice showed no differences from controls in freezing during habituation (t-test, p=0.82) or in social fear learning (t-test, p=0.33). Based on these results, fear recall in classic fear conditioning was selected as the primary outcome measure of interest for the replication cohort, with no social fear transmission component.

*Operant response training.* DPE mice in the discovery cohort showed a trend toward more failures to pass acquisition criterion (chi-squared test, p=0.09; Table S1). There were no differences between exposures in the 7-day or the 15-day acquisition curves (ANOVAs, no main effects of exposure or exposure x day interactions). The FR1 acquisition criterion (chi-squared test) was chosen as the primary outcome measure of interest for the replication cohort.

*Contingency degradation tests.* Following each of the three training phases, subjects that successfully learned the task were tested on response-outcome contingency degradation (Table S1). Following the initial FR1 training, control mice preferred the active aperture in the CD test (t-test, p=0.04), and this preference gradually degraded following overtraining sessions (t-tests, p>0.1), a pattern typical of gradual habit formation in healthy controls. DPE mice showed no preference for the active aperture in the first CD test (t-test, p>0.1) and gradually acquired a preference in later tests (t-tests, CD2, p=0.022; CD3, p=0.004), a pattern of preference that differed significantly from controls (ANOVA, exposure x epoch x aperture interaction, F(2,18)=5.9, p=0.011; post-hoc t-tests, CD1, p=0.042; CD2, p=0.94; CD3, p=0.026). This pattern of responding in DPE mice suggests a cognitive impairment in re-acquiring new contingencies ^6^. Nonetheless, because subjects from the DPE treatment group were preferentially eliminated in FR1 training for failure to acquire the task, contingency degradation outcome measures were not selected for replication.

**Supplementary References**

1 Caruso, A., Ricceri, L. & Scattoni, M. L. Ultrasonic vocalizations as a fundamental tool for early and adult behavioral phenotyping of Autism Spectrum Disorder rodent models. *Neurosci Biobehav Rev* **116**, 31-43, doi:10.1016/j.neubiorev.2020.06.011 (2020)

2 McFarlane, H. G. *et al.* Autism‐like behavioral phenotypes in BTBR T+ tf/J mice. *Genes, Brain and Behavior* **7**, 152-163 (2008)

3 Swanson, A. M., DePoy, L. M. & Gourley, S. L. Inhibiting Rho kinase promotes goal-directed decision making and blocks habitual responding for cocaine. *Nature communications* **8**, 1-12 (2017)

4 DePoy, L. M., Zimmermann, K. S., Marvar, P. J. & Gourley, S. L. Induction and blockade of adolescent cocaine-induced habits. *Biological psychiatry* **81**, 595-605 (2017)

5 Richardson, J. R. *et al.* Developmental pesticide exposure reproduces features of attention deficit hyperactivity disorder. *The FASEB Journal* **29**, 1960-1972 (2015)

6 Gross, C. *et al.* Selective role of the catalytic PI3K subunit p110β in impaired higher order cognition in fragile X syndrome. *Cell reports* **11**, 681-688 (2015)

| Table S1. Discovery cohorts. | | | | | | |
| --- | --- | --- | --- | --- | --- | --- |
| Assays | Measures | Statistical test | Test statistic | p-value | Sig. | Effect Size |
| Separation-induced USVs | Call number | ANOVA, main effect of exposure | F(1,6)=16.7 | p=0.006 | *** | e=0.74 |
|  |  | ANOVA, main effect of session | F(1,6)=67.5 | p<0.0005 | *** | e=0.92 |
|  |  | ANOVA, exposure x sex interaction | F(1,6)=0.0 | p=1.0 |  | e=0 |
|  |  | ANOVA, exposure x session interaction | F(1,6)=0.47 | p=0.52 |  | e=0.073 |
|  |  | ANOVA, exposure x sex x session interaction | F(1,6)=1.5 | p=0.27 |  | e=0.19 |
| Separation-induced USVs | Call features | MANOVA, main effect of exposure | F(1,6)=0.078 | p=0.79 |  | e=0.013 |
|  |  | MANOVA, exposure x sex interaction | F(1,6)=0.59 | p=0.47 |  | e=0.089 |
|  | Call features (male) | MANOVA, exposure x mean frequency | F(1,6)=0.44 | p=0.53 |  | e=0.068 |
|  |  | MANOVA, exposure x median frequency | F(1,6)=0.57 | p=0.48 |  | e=0.087 |
|  |  | MANOVA, exposure x max frequency | F(1,6)=0.43 | p=0.54 |  | e=0.067 |
|  |  | MANOVA, exposure x min frequency | F(1,6)=3.5 | p=0.11 |  | e=0.37 |
|  |  | MANOVA, exposure x long frequency | F(1,6)=0.72 | p=0.43 |  | e=0.11 |
|  |  | MANOVA, exposure x loud frequency | F(1,6)=0.46 | p=0.52 |  | e=0.071 |
|  |  | MANOVA, exposure x mean amplitude | F(1,6)=1.3 | p=0.29 |  | e=0.18 |
|  |  | MANOVA, exposure x median amplitude | F(1,6)=1.1 | p=0.33 |  | e=0.16 |
|  |  | MANOVA, exposure x max amplitude | F(1,6)=0.62 | p=0.46 |  | e=0.093 |
|  |  | MANOVA, exposure x min amplitude | F(1,6)=3.2 | p=0.12 |  | e=0.35 |
|  |  | MANOVA, exposure x percnan | F(1,6)=0.081 | p=0.79 |  | e=0.013 |
|  |  | MANOVA, exposure x jump call # | F(1,6)=0.027 | p=0.87 |  | e=0.005 |
|  |  | MANOVA, exposure x jump call % | F(1,6)=0.006 | p=0.94 |  | e=0.001 |
|  |  | MANOVA, exposure x start frequency | F(1,6)=1.3 | p=0.30 |  | e=0.17 |
|  |  | MANOVA, exposure x start derivative | F(1,6)=2.5 | p=0.17 |  | e=0.29 |
|  |  | MANOVA, exposure x median derivative | F(1,6)=2.3 | p=0.18 |  | e=0.28 |
|  |  | MANOVA, exposure x max abs derivatuve | F(1,6)=0.21 | p=0.66 |  | e=0.034 |
|  |  | MANOVA, exposure x max derivative limit zero | F(1,6)=0.99 | p=0.36 |  | e=0.14 |
|  |  | MANOVA, exposure x bandwidth frequency | F(1,6)=0.45 | p=0.53 |  | e=0.07 |
|  |  | MANOVA, exposure x end frequency | F(1,6)=0.015 | p=0.91 |  | e=0.002 |
|  | Call features (female) | MANOVA, exposure x mean frequency | F(1,6)=0.50 | p=0.51 |  | e=0.076 |
|  |  | MANOVA, exposure x median frequency | F(1,6)=0.69 | p=0.44 |  | e=0.10 |
|  |  | MANOVA, exposure x max frequency | F(1,6)=0.037 | p=0.85 |  | e=0.006 |
|  |  | MANOVA, exposure x min frequency | F(1,6)=1.9 | p=0.21 |  | e=0.24 |
|  |  | MANOVA, exposure x long frequency | F(1,6)=0.32 | p=0.59 |  | e=0.05 |
|  |  | MANOVA, exposure x loud frequency | F(1,6)=0.34 | p=0.58 |  | e=0.053 |
|  |  | MANOVA, exposure x mean amplitude | F(1,6)=2.4 | p=0.18 |  | e=0.28 |
|  |  | MANOVA, exposure x median amplitude | F(1,6)=3.0 | p=0.13 |  | e=0.33 |
|  |  | MANOVA, exposure x max amplitude | F(1,6)=0.34 | p=0.58 |  | e=0.053 |
|  |  | MANOVA, exposure x min amplitude | F(1,6)=0.39 | p=0.56 |  | e=0.061 |
|  |  | MANOVA, exposure x percnan | F(1,6)=4.0 | p=0.092 |  | e=0.4 |
|  |  | MANOVA, exposure x jump call # | F(1,6)=0.36 | p=0.57 |  | e=0.056 |
|  |  | MANOVA, exposure x jump call % | F(1,6)=2.5 | p=0.16 |  | e=0.30 |
|  |  | MANOVA, exposure x start frequency | F(1,6)=1.8 | p=0.23 |  | e=0.23 |
|  |  | MANOVA, exposure x start derivative | F(1,6)=3.5 | p=0.11 |  | e=0.37 |
|  |  | MANOVA, exposure x median derivative | F(1,6)=0.76 | p=0.42 |  | e=0.11 |
|  |  | MANOVA, exposure x max abs derivatuve | F(1,6)=0.38 | p=0.56 |  | e=0.06 |
|  |  | MANOVA, exposure x max derivative limit zero | F(1,6)=0.60 | p=0.47 |  | e=0.09 |
|  |  | MANOVA, exposure x bandwidth frequency | F(1,6)=0.13 | p=0.73 |  | e=0.021 |
|  |  | MANOVA, exposure x end frequency | F(1,6)=0.01 | p=0.92 |  | e=0.002 |
| Juvenile social interaction | Social behaviors | MANOVA, main effect of exposure | F(2,6)=0.52 | p=0.77 |  | e=0.61 |
|  |  | MANOVA, exposure x self-grooming | F(1,7)=0.011 | p=0.92 |  | e=0.002 |
|  |  | MANOVA, exposure x following | F(1,7)=0.0 | p=0.99 |  | e<0.0005 |
|  |  | MANOVA, exposure x face sniffing | F(1,6)=0.38 | p=0.56 |  | e=0.052 |
|  |  | MANOVA, exposure x body/genital sniffing | F(1,6)=0.99 | p=0.35 |  | e=0.12 |
|  |  | MANOVA, exposure x allogrooming | F(1,6)=0.086 | p=0.78 |  | e=0.012 |
|  |  | MANOVA, exposure x huddling | F(1,6)=1.5 | p=0.27 |  | e=0.17 |
| Adult novel cage observation | Repetitive behaviors | MANOVA on ranks, main effect of exposure | F(3,5)=3.3 | p=0.11 |  | e=0.69 |
|  |  | MANOVA on ranks, exposure x self-grooming | F(1,7)=10.8 | p=0.013 | * | e=0.61 |
|  |  | MANOVA on ranks, exposure x digging | F(1,7)=0.22 | p=0.66 |  | e=0.03 |
|  |  | MANOVA on ranks, exposure x inchworming | F(1,7)=0.22 | p=0.66 |  | e=0.03 |
| Adult social interaction | Social behaviors | MANOVA on ranks, main effect of exposure | F(4,4)=3.3 | p=0.14 |  | e=0.76 |
|  |  | MANOVA on ranks, exposure x self-grooming | F(1,7)=0.96 | p=0.36 |  | e=0.12 |
|  |  | MANOVA on ranks, exposure x following | F(1,7)=4.3 | p=0.076 | # | e=0.38 |
|  |  | MANOVA on ranks, exposure x face sniffing | F(1,7)=0.96 | p=0.36 |  | e=0.12 |
|  |  | MANOVA on ranks, exposure x body/genital sniffing | F(1,7)=0.96 | p=0.36 |  | e=0.12 |
|  |  | MANOVA, exposure x allogrooming | NA | NA |  | NA |
|  |  | MANOVA, exposure x huddling | NA | NA |  | NA |
| Marble burying | Marbles buried | T-test, control vs. DPE | t=-5.0 | p=0.002 | *** | d=3.3 |
| Three-chamber social approach |  | ANOVA, main effect of exposure | F(1,7)=0.64 | p=0.45 |  | e=0.083 |
|  |  | ANOVA, main effect of stimulus | F(3,21)=5.5 | p=0.006 | *** | e=0.45 |
|  |  | ANOVA, exposure x stimulus interaction | F(3,21)=0.080 | p=0.97 |  | e=0.011 |
|  |  | T-test, all groups, object vs. mouse | t=-3.2 | p=0.012 | * | d=-1.1 |
|  |  | T-test, all groups, familiar vs. novel | t=-2.0 | p=0.086 | # | d=0.62 |
| Operant conditioning | Acquisition criterion | Chi-squared | χ2 = 2.9 | p=0.09 | # | e=0.18 |
|  | 7-day acquisition curve | ANOVA, main effect of day | F(6,84)=9.4 | p<0.0005 | *** | e=0.39 |
|  |  | ANOVA, main effect of exposure | F(1,14)=1.6 | p=0.23 |  | e=0.10 |
|  |  | ANOVA, exposure x day interaction | F(6,84)=1.1 | p=0.36 |  | e=0.052 |
|  | 15-day acquisition curve | ANOVA, main effect of day | F(14,126)=33.5 | p<0.0005 | *** | e=0.79 |
|  |  | ANOVA, main effect of exposure | F(1,9)=0.31 | p=0.59 |  | e=0.033 |
|  |  | ANOVA, exposure x day interaction | F(14,126)=0.85 | p=0.61 |  | e=0.085 |
| Instrumental contingency degradation | Aperture preference | ANOVA, main effect of exposure | F(1,9)=9.8 | p=0.012 | * | e=0.52 |
|  |  | ANOVA, exposure x epoch x aperture interaction | F(2,18)=5.9 | p=0.011 | * | e=0.39 |
|  |  | T-test, CD1, control, active vs. degraded | t=2.8 | p=0.04 | * | d=1.1 |
|  |  | T-test, CD1, DPE, active vs. degraded | t=-0.14 | p=0.90 |  | d=-0.61 |
|  |  | T-test, CD2, control, active vs. degraded | t=1.4 | p=0.21 |  | d=0.59 |
|  |  | T-test, CD2, DPE, active vs. degraded | t=3.6 | p=0.022 | * | d=1.6 |
|  |  | T-test, CD3, control, active vs. degraded | t<0.0005 | p=1.0 |  | d=0.000 |
|  |  | T-test, CD3, DPE, active vs. degraded | t=6.1 | p=0.004 | *** | d=2.7 |
|  |  | T-test, CD1, preference, control vs. DPE | t=2.4 | p=0.042 | * | d=1.4 |
|  |  | T-test, CD2, preference, control vs. DPE | t=-0.79 | p=0.94 |  | d=-0.048 |
|  |  | T-test, CD3, preference, control vs. DPE | t=-2.7 | p=0.026 | * | d=-1.6 |
| Classical fear conditioning | Baseline freezing | T-test, control vs. DPE | t=4.1 | p=0.004 | *** | d=2.8 |
|  | Fear acquisition | ANOVA, main effect of tone | F(4,28)=20.9 | p<0.0005 | *** | e=0.75 |
|  |  | ANOVA, main effect of exposure | F(1,7)=2.2 | p=0.18 |  | e=0.24 |
|  |  | ANOVA, time x exposure interaction | F(4,28)=5.5 | p=0.002 | *** | e=0.44 |
|  | Habituation to Context B | T-test, control vs. DPE | t=0.56 | p=0.59 |  | d=0.38 |
|  | Fear expression | T-test, control vs. DPE | t=2.4 | p=0.045 | * | d=1.6 |
| Social fear transmission | Habituation to Context B | T-test, control vs. DPE | t=0.022 | p=0.983 |  | d=0.015 |
|  | Fear expression | T-test, control vs. DPE | t=-0.67 | p=0.52 |  | d=-0.45 |

Table S1. Statistical results from all measures and statistical tests in the discovery cohorts. Effect sizes are in partial eta squared (e) or Cohen’s d (d).

| Table S2. Replication cohort. | | | | | | | | | | |
| --- | --- | --- | --- | --- | --- | --- | --- | --- | --- | --- |
| Assays | Measures | Statistical test | Test statistic | p-value | sig | Effect size | Combined test | Combined p | sig | Combined effect size |
| Separation-induced USVs | Call number | ANOVA, main effect of exposure | F(1,29)=0.19 | p=0.67 |  | e=0.006 | F(1,36)=0.42 | p=0.52 |  | e=0.012 |
|  |  | ANOVA, exposure x sex interaction | F(1,29)=4.5 | p=0.043 | * | e=0.13 | F(1,36)=4.3 | p=0.045 | * | e=0.11 |
|  |  | T-test, male, control vs. DPE | t=-1.7 | p=0.048 | * | d=0.62 | F(1,36)=4.7 | p=0.036 | * | e=0.12 |
|  |  | T-test, female, control vs. DPE | t=1.2 | p=0.12 |  | d=-0.44 | F(1,36)=0.036 | p=0.85 |  | e=0.001 |
| Classical fear conditioning | Baseline freezing | T-test, control vs. DPE | t=0.53 | p=0.60 |  | d=0.19 | F(1,39)=0.083 | p=0.77 |  | e=0.002 |
|  | Fear acquisition | ANOVA, main effect of tone | F(4,124)=85.5 | p<0.0005 | *** | e=0.73 | F(4,156)=11.6 | p<0.0005 | *** | e=0.23 |
|  |  | ANOVA, main effect of exposure | F(1,31)=0.030 | p=0.86 |  | e=0.001 | F(1,39)=0.21 | p=0.65 |  | e=0.005 |
|  |  | ANOVA, tone x exposure interaction | F(4,124)=0.24 | p=0.92 |  | e=0.0086 | F(4,156)=1.5 | p=0.20 |  | e=0.038 |
|  | Fear recall | T-test, control vs. DPE | t=-1.9 | p=0.036 | * | d=-0.65 | F(1,39)=7.9 | p=0.008 | * | e=0.17 |
| Operant conditioning | Acquisition criterion | Chi-squared test | χ2=4.0 | p=0.022 | * | e=0.13 | χ2=6.9 | p=0.0042 | ** | e=0.14 |
|  | 7-day acquisition curve | ANOVA, main effect of day | F(6,180)=28.2 | p<0.0005 | *** | e=0.49 | f(6,270)=12.2 | p<0.0005 | *** | e=0.21 |
|  |  | ANOVA, main effect of exposure | F(1,30)=1.3 | p=0.27 |  | e=0.040 | F(1,45)=2.6 | p=0.11 |  | e=0.055 |
|  |  | ANOVA, exposure x day interaction | F(6,180)=1.3 | p=0.27 |  | e=0.041 | F(6,270)=1.5 | p=0.18 |  | e=0.032 |
| Marble burying | Marbles buried | T-test, control vs. DPE | t=2.2 | p=0.20 | * | d=0.76 | F(1,39)=7.2 | p=0.011 | * | e=0.16 |
| Adult novel cage observation | Repetitive behaviors | T-test, self-grooming, control vs. DPE | t=-2.7 | p=0.006 | * | d=-0.96 | F(1,39)=10.2 | p=0.003 | ** | e=0.21 |

Table S2. Statistical results from all measures and statistical tests in the discovery cohorts. Effect sizes are in partial eta squared (e) or Cohen’s d (d). All combined tests are ANCOVAs.

| Table S3. Biological measures. | | | | | | |
| --- | --- | --- | --- | --- | --- | --- |
| Assays | Measures | Statistical test | Test statistic | p-value | sig | Effect size |
| Fast-scan cyclic voltammetry | Peak striatal dopamine release (dorsal) | t-test | t=-2.3 | p=0.037 | * | d=-1.3 |
|  | Peak striatal dopamine release (ventral) | t-test | t=-2.3 | p=0.041 | * | d=-1.2 |
|  | Tau (dorsal) | t-test | t=-0.38 | p=0.71 |  | d=-0.21 |
|  | Tau (ventral) | t-test | t=-0.20 | p=0.84 |  | d=-0.11 |
| HPLC | Striatal dopamine per unit protein | t-test | t=-2.9 | p=0.011 | * | d=-1.5 |
|  | Striatal DOPAC per unit protein | t-test | t=-0.90 | p=0.38 |  | d=-0.46 |
|  | Striatal HVA per unit protein | t-test | t=-3.2 | p=0.0063 | * | d=-1.6 |
|  | Striatal 3-MT per unit protein | t-test | t=-2.6 | p=0.019 | * | d=-1.3 |
| Vesicular loading assay | [3H]dopamine uptake | t-test | t=-0.79 | p=0.44 |  | d=-.32 |
| Whole cell patch clamp | Cell properties | MANOVA, main effect of exposure | F(1,13)=2.2 | p=0.037 | * | e=0.47 |
|  |  | MANOVA, exposure x RMP | F(1,44)=0.52 | p=0.48 |  | e=0.012 |
|  |  | MANOVA, exposure x IH ratio | F(1,44)=2.5 | p=0.12 |  | e=0.053 |
|  |  | MANOVA, exposure x IK(IR) ratio | F(1,44)=4.7 | p=0.035 | * | e=0.097 |
|  |  | MANOVA, exposure x Tau (ms) | F(1,44)=3.5 | p=0.07 | # | e=0.073 |
|  |  | MANOVA, exposure x RIN (MΩ) | F(1,44)=1.04 | p=0.31 |  | e=0.023 |
|  |  | MANOVA, exposure x AP amplitude (mV) | F(1,44)=0.73 | p=0.40 |  | e=0.016 |
|  |  | MANOVA, exposure x AP half-width (ms) | F(1,44)=6.02 | p=0.018 | * | e=0.12 |
|  |  | MANOVA, exposure x AP rise time 10-90% (ms) | F(1,44)=3.07 | p=0.087 | # | e=0.065 |
|  |  | MANOVA, exposure x AP decay time 90-10% (ms) | F(1,44)=5.8 | p=0.020 | * | e=0.12 |
|  |  | MANOVA, exposure x AP threshold | F(1,44)=0.30 | p=0.59 |  | e=0.007 |
|  |  | MANOVA, exposure x ISI 1 (ms) | F(1,44)=1.2 | p=0.29 |  | e=0.026 |
|  |  | MANOVA, exposure x ISIN (ms) | F(1,44)=0.12 | p=0.75 |  | e=0.002 |
|  |  | MANOVA, exposure x ISI 1/ISIN | F(1,44)=0.39 | p=0.53 |  | e=0.009 |
|  |  | MANOVA, exposure x fAHP (ms) | F(1,44)=0.60 | p=0.44 |  | e=0.013 |

Table S3. Statistical results from all biological measures and statistical tests. Effect sizes are in partial eta squared (e) or Cohen’s d (d).

Figure S1. Vesicular dopamine uptake in striatal vesicles.


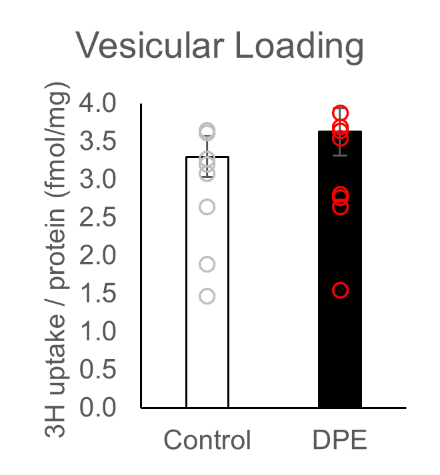


**Figure 3.** Vesicular dopamine capacity in striatal vesicles. Dopamine uptake into striatal vesicles was not different between exposure groups (N=12 per group).
